# Study of the optimum haplotype length to build genomic relationship matrices

**DOI:** 10.1186/s12711-016-0253-6

**Published:** 2016-09-29

**Authors:** Mohammad H. Ferdosi, John Henshall, Bruce Tier

**Affiliations:** 1 The Centre for Genetic Analysis and Applications, School of Environmental and Rural Science, University of New England, Armidale, Australia; 2 Animal Genetics and Breeding Unit, University of New England, Armidale, Australia; 3 Cobb-Vantress, Siloam Springs, AR USA; 4 CSIRO Agriculture Flagship, FD McMaster Laboratory Chiswick, Armidale, Australia

## Abstract

**Background:**

As genomic data becomes more abundant, genomic prediction is more routinely used to estimate breeding values. In genomic prediction, the relationship matrix (${\mathbf{A}}$), which is traditionally used in genetic evaluations is replaced by the genomic relationship matrix (${\mathbf{G}}$). This paper considers alternative ways of building relationship matrices either using single markers or haplotypes of different lengths. We compared the prediction accuracies and log-likelihoods when using these alternative relationship matrices and the traditional ${\mathbf{G}}$ matrix, for real and simulated data.

**Methods:**

For real data, we built relationship matrices using 50k genotype data for a population of Brahman cattle to analyze three traits: scrotal circumference (SC), age at puberty (AGECL) and weight at first corpus luteum (WTCL). Haplotypes were phased with hsphase and imputed with BEAGLE. The relationship matrices were built using three methods based on haplotypes of different lengths. The log-likelihood was considered to define the optimum haplotype lengths for each trait and each haplotype-based relationship matrix.

**Results:**

Based on simulated data, we showed that the inverse of ${\mathbf{G}}$ matrix and the inverse of the haplotype relationship matrices for methods using one-single nucleotide polymorphism (SNP) phased haplotypes provided coefficients of determination (R^2^) close to 1, although the estimated genetic variances differed across methods. Using real data and multiple SNPs in the haplotype segments to build the relationship matrices provided better results than the ${\mathbf{G}}$ matrix based on one-SNP haplotypes. However, the optimal haplotype length to achieve the highest log-likelihood depended on the method used and the trait. The optimal haplotype length (7 to 8 SNPs) was similar for SC and AGECL. One of the haplotype-based methods achieved the largest increase in log-likelihood for SC, i.e. from −1330 when using ${\mathbf{G}}$ to −1325 when using haplotypes with eight SNPs.

**Conclusions:**

Building the relationship matrix by using haplotypes that comprise multiple SNPs will increase the accuracy of estimated breeding values. However, the optimum haplotype length that shows the correct relationship among individuals for each trait can be derived from the data.

## Background

Advances in genotyping technologies have resulted in a substantial decrease in genotyping costs for many species. These advances have created a new era in livestock genetic evaluation by adding a new type of information to the traditional animal breeding techniques. In the past, genetic evaluation was based on phenotypic records and pedigree information with best linear unbiased prediction (BLUP) [1]. In spite of the complexity of the underlying biology, traditional genetic evaluation methods have had a large effect on the improvement of livestock production. Usually an animal model was used, i.e. a model that includes each animal’s breeding value where the numerator relationship matrix (${\mathbf{A}}$) was used to define the genetic relationships among animals. Relationships in ${\mathbf{A}}$ are twice the co-ancestry of pairs of individuals and ${\mathbf{A}}$ is built by tracking the descent of founder genomes (from the base population, i.e. animals whose pedigrees are unknown and are assumed to be unrelated [2]) through the pedigree. Thus, elements in ${\mathbf{A}}$ are based on the idea of identity by descent (IBD).

The availability of cheap genomic data in large quantities allows the relationships among individuals to be defined directly and thus, more accurately. This led to the development of the genomic relationship matrix (${\mathbf{G}}$) [3–7], which can replace ${\mathbf{A}}$ in genetic evaluations. ${\mathbf{G}}$ was designed for use with large numbers of independent SNPs (single nucleotide polymorphisms).

Unlike ${\mathbf{A}}$, ${\mathbf{G}}$ reflects identity by state (IBS) within the population, and thus relationships arise between pairs of individuals that were previously considered ‘unrelated’. Hence ${\mathbf{G}}$ incorporates relationships that arise from unknown common ancestors. These ancestors predate the animals that are considered to be founders by pedigree information. Using ${\mathbf{G}}$ in place of ${\mathbf{A}}$ can increase the accuracy of parameter estimation and decrease the expense of progeny-testing [3, 8]. Furthermore, ${\mathbf{G}}$ allows us to estimate the breeding value for new individuals more accurately than ${\mathbf{A}}$ by using only the genotype data and the phenotype of ancestral individuals [9].

${\mathbf{G}}$ considers complete linkage disequilibrium (LD) between SNPs and quantitative trait loci (QTL) but ignores LD between SNPs, especially in short regions [10]. Therefore, shuffling the order of SNPs has no effect on the final results in ${\mathbf{G}}$. However, a desirable QTL allele in one sub-population may be in LD with the allele of one SNP in one strand of the haplotype, but with the other allele of the SNP in another haplotype in the other part of the population. Capturing this type of variable phase between SNPs and QTL requires using a group of SNPs that are joined together in the form of haplotypes [11].

Livestock populations usually consist of large numbers of half-sib and full-sib families. This population structure allows us to reconstruct (phase) the haplotypes accurately and rapidly [12, 13]. Combining IBD and LD information to describe relationships by using haplotypes can increase the accuracy of genetic evaluation and parameter estimation [4, 11, 14, 15]. Hickey et al. [15] used regional haplotype information (non-overlapping haplotype segments, i.e. distinct windows) by breaking haplotypes of all individuals into short segments of equal size (5 to 2000 SNPs) to estimate the relationships between individuals for each segment. The average relationships among all segments were calculated to estimate the total relationship between individuals using simulated data. As reported by Hickey et al. [15], this new method did not improve accuracy of prediction compared to the method based on unphased genotypes. However, they found a higher correlation between their diagonal and off-diagonal elements of the relationship matrix and the true relationship matrix (simulated data), than between those of the ${\mathbf{G}}$ matrix generated from individual SNPs. Simulated data may not represent the real populations’ genotypes and phenotypes because of the underlying biological complexity. The partitioning of the genome, as suggested by Hickey et al. [15], in non-overlapping haplotype segments [distinct windows (DW)] may not capture all the variation of the haplotype diversity across the entire genome, because the linkage between some haplotype segments may be ignored. This linkage can be accounted for, by partitioning the genome into segments that overlap (sliding windows (SW) [16]).

The challenge in choosing the optimal haplotype length is to model linkage between SNPs and QTL appropriately. When haplotypes are based on one-SNP (individual SNPs), there are only two possible alleles. When haplotypes are based on pairs of SNPs, four alleles are possible and as the number of SNPs in each segment increases, so does the number of possible alleles. Varying the length of haplotypes can assist in the modeling of the LD between SNPs and QTL. On the one hand, if haplotype similarity is the basis of determining relationships, increasing the number of haplotype alleles will generally result in lower relationships within any segment. On the other hand, using short haplotype segments will maintain remote relationships within the matrix, but may also capture different phases between SNPs and QTL by ignoring LD. The optimal haplotype length will balance the value of older relationships against the errors in LD that are assumed between SNPs and QTL across the whole population [4].

The aim of this study was to choose the best relationship matrix based on different ways of modeling LD between SNPs and QTL and identifying the optimal haplotype length. Three alternative relationship matrices, based on haplotypes of variable length, were considered and compared with the standard ${\mathbf{G}}$ matrix [3] for three traits. The underlying ideas in the construction of ${\mathbf{G}}$ and these alternative methods were explored with real and simulated data.

## Methods

### Data

#### Simulated data

A small dataset was simulated for the purpose of exploring ${\mathbf{G}}$ (VanRaden [3]—first method) when considering whether to include or exclude allele coding and allele frequencies on ${\mathbf{G}}$ and its inverse. In addition, our objective was to understand the effects of allele coding of the genotype and correcting allele frequencies in order to build ${\mathbf{G}}$ on the log-likelihood and variance components. Since the simulated ${\mathbf{G}}$ was based on one-SNP haplotypes, LD between markers was ignored. This dataset was based on a full-sib design of four males each mated to five females to produce one offspring per mating. The final population included nine parents (four males and five females) and 20 offspring. A trait with a heritability of 0.55 and 99 SNPs was simulated. The phenotypes were simulated as:

1 $${\text{Phenotype}} = {\mathbf{qX}}_{\text{G}} + {\mathbf{e}} ,$$

where ${\mathbf{q}}$ is a vector of SNP effects N(0,1), ${\mathbf{X}}_{\text{G}}$ is a genotype matrix with terms equal to the genotypes (defined as the number (0, 1, or 2) of second alleles of each animal at each SNP) and ${\mathbf{e}}$ is a vector of normally-distributed residuals. Genotypes were simulated at the gametic level with equally-spaced SNPs on 10 chromosomes, each 1 Morgan long.

#### Real data

A subset of the 50k SNP data obtained from the “Northern Breeding Project” resource Brahman population bred by the Cooperative Research Centre for Beef Genetic Technologies (BeefCRC) was used, with trait records for scrotal circumference (SC), age at puberty (AGECL), and weight at first corpus luteum (WTCL). The description and details of SC, WTCL and AGECL phenotypes were provided by Johnston et al. [17], Hawken et al. [18] and Zhang et al. [19]. Estimation of heritabilities was based on the single-trait animal model using ${\mathbf{A}}$ with the following fixed effects for each trait (see comments in Table 1):

**Table 1:** Number of animals (N), mean (${\varvec{\upmu}}$), standard deviation (SD) and heritability (h^2^) for different traits [17–19]

Trait	N	µ	SD	h^2^
SC (cm)	1007	26.6	2.94	0.75^a^
AGECL (days)	854	751	142.1	0.57^a^
WTCL (kg)	854	334	44.8	0.56^a^

*SC* scrotal circumference,* AGECL* age at puberty,* WTCL* weight at first corpus luteum

^a^BLUP using matrix ${\text{A}}$

- SC: cohort, location, month of birth, operator, age and weight at 18 months;
- AGECL: age of dam, cohort, origin, calving month, interaction of origin and calving month, interaction of cohort and origin, interaction of cohort and calving month;
- WTCL: age of dam, cohort, line of origin and calving month.

#### Ethical approval

This experiment was approved by the JM. Rendel Laboratory Animal Experimental Ethics Committee (CSIRO, Queensland) as approvals TBC107 and RH225-06, respectively.

### Haplotypes

Overall haplotypes for the Brahman cattle were reconstructed for all the chromosomes using hsphase [12, 13] and missing genotypes were imputed by BEAGLE 3.3.2 [20]. hsphase and BEAGLE were run with default parameters. The whole genome was subsequently divided into segments of equal length (1, 2, 3, …, 20, 40, 80 and 100) and the numbers of haplotype alleles in each segment were identified.

### Relationship matrices

The following relationship matrices were built to determine the effect of centering and correcting allele frequency on the additive and residual variances.

#### Genomic relationship matrix using independent SNPs (centered)

In our work, ${\mathbf{G}}$ refers to VanRaden’s first method [3] for calculating the genetic relationship matrix. To construct ${\mathbf{G}}$ in a population with ‘a’ animals genotyped for ‘m’ SNPs, the genotypes were centered so that the sum of each column was zero, ${\mathbf{Z}} = {\mathbf{X}}_{{\mathbf{G}}} - {\mathbf{P}} = {\mathbf{X}}_{{\mathbf{G}}} - {\mathbf{J}} - {\mathbf{D}}$, where ${\mathbf{X}}_{{\mathbf{G}}}$ is the genotype matrix (a × m), with entries 0, 1 or 2, representing alleles AA, AB and BB, respectively; ${\mathbf{D}} = {\mathbf{P}} - {\mathbf{J}}$, where ${\mathbf{P}}$ is an (${\text{a}} \times {\text{m}}$) matrix with each row consisting of 2**p** ($\mathbf{p}$ is the **B** allele frequency of each SNP) and ${\mathbf{J}}$ is a matrix of 1’s with the same dimension as ${\mathbf{P}}$. Finally, ${\mathbf{G}}$ was calculated as:

2 $${\mathbf{G}} = \frac{{{\mathbf{ZZ}}^{\prime } }}{{2\sum\mathbf{p}\left( {1 - \mathbf{p}} \right)}},$$

Because ${\mathbf{G}}$ was not positive definite, 0.001 was added to its diagonal elements, to allow inversion.

#### Genetic relationship matrix using independent SNPs (uncentered)

A matrix ${\mathbf{M}}$ was constructed from ${\mathbf{X}}_{{\mathbf{G}}}$ by subtracting **1**, via ${\mathbf{M}} = {\mathbf{X}}_{{\mathbf{G}}} - {\mathbf{J}}$. This matrix included 1, 0 and −1 representing alleles AA, AB and BB.

Matrix ${\mathbf{M}}$ was used to calculate a matrix that is similar to ${\mathbf{G}}$ but uncentered for the allele frequencies (${\mathbf{G}}_{\text{u}}$):

3 $${\mathbf{G}}_{\text{u}} = \frac{{{\mathbf{MM}}^{\prime } }}{{{\text{d}} }},$$

where the denominator $\textrm{d} = \textrm{m}/{{2}}$ = ${2\sum\mathbf{p}\left( {1 - \mathbf{p}} \right)}$, assuming ${\textbf{p}} = {0.5}$. ${\mathbf{G}}_{{\mathbf{u}}}$ was used to demonstrate the effect of centering on additive and residual variances. Alternatively, the same denominator that is used in ${\mathbf{G}}$ (i.e. calculating allele frequency after centering) could be used.

#### Relationship matrices using one-SNP haplotypes

Haplotypes of animals were used to create the one-SNP haplotype relationship matrix. Let ${\mathbf{X}}_{\text{H}}$ be a (${\text{h }} \times {\text{m}}$) matrix of haplotypes (${\text{h }} = 2{\text{a}}$), with entries 0 or 1 indicating the number of copies of one of the two possible alleles. For a single locus, haplotypes were constructed without reference to the adjacent loci. Suppose that ${\mathbf{K}} = {\mathbf{I}}_{\text{ah}} \otimes \left[ {1\;1} \right]$ (${\mathbf{I}}$ is an identity matrix, and $\otimes$ is the Kronecker product [21]). With ${\mathbf{X}}_{\text{H}}$ and ${\mathbf{K}}$, the genotypes were reconstructed as ${\mathbf{X}}_{\text{G}} = {\mathbf{KX}}_{\text{H}}$. The allele frequencies for SNP in ${\mathbf{X}}_{\text{H}}$ were calculated as $\mathbf{p} = 1{\mathbf{X}}_{\text{H}} /{\mathbf{h}}$.

The haplotype relationship matrix for one-SNP (${\mathbf{H}}_{*,1} )$ can be calculated as follows:

4 $${\mathbf{H}}_{*,1} = {\mathbf{K}}{\varvec{\Gamma}} {\mathbf{K}}^{{\prime }} /2,$$

such that:

5 $${{\varvec{\Gamma}}} = ({\mathbf{X}}_{\text{H}} {\mathbf{X}}_{\text{H}}^{\prime } + ({\mathbf{X}}_{\text{H}} - {\mathbf{J}}_{\text{hm}} )({\mathbf{X}}_{\text{H}} - {\mathbf{J}}_{\text{hm}} )^{{\prime }} )/{\text{m}} .$$

Alternatively ${{\varvec{\Gamma}}}$ can be computed as:

6 $${{\varvec{\Gamma}}} = \left( {{\mathbf{J}}_{\text{hm}} {\mathbf{J}}_{\text{hm}}^{\prime } - \left( {{\mathbf{Q}} + {\mathbf{Q}}^{\prime } } \right)} \right)/{\text{m}} ,$$

where ${\mathbf{Q}}$ is ${\mathbf{X}}_{\text{H}} \left( { - \left( {{\mathbf{X}}_{\text{H}} - {\mathbf{J}}_{\text{hm}} } \right)^{{\prime }} } \right)$, which is similar to the method explained in [22].

#### Similarity of ${\mathbf{G}}$ and ${\mathbf{H}}_{*,1}$

Expansion of the terms for the ${\mathbf{G}}$ (7), ${\mathbf{G}}_{\text{u}}$ (10) and ${\mathbf{H}}_{*,1}$ (11) matrices helps to illustrate the differences between them.

7 $${\mathbf{G}} = {\mathbf{ZZ}}^{\prime } /{\text{d}} = \left( {\left( {{\mathbf{X}}_{\text{G}} - {\mathbf{J}}_{\text{am}} - {\mathbf{D}}} \right)\left( {{\mathbf{X}}_{\text{G}} - {\mathbf{J}}_{\text{am}} - {\mathbf{D}}} \right)^{\prime } } \right)/{\text{d}} ,$$

$${\mathbf{G}} = \left( {{\mathbf{X}}_{\text{G}} {\mathbf{X}}_{\text{G}}^{'} - {\mathbf{X}}_{\text{G}} {\mathbf{J}}_{\text{am}}^{'} - {\mathbf{X}}_{\text{G}} {\mathbf{D^{\prime}}} - {\mathbf{J}}_{\text{am}} {\mathbf{X}}_{\text{G}}^{'} + {\mathbf{J}}_{\text{am}} {\mathbf{J}}_{\text{am}}^{'} + {\mathbf{J}}_{\text{am}} {\mathbf{D^{\prime}}} - {\mathbf{DX}}_{\text{G}}^{'} + {\mathbf{DJ}}_{\text{am}}^{'} + {\mathbf{DD^{\prime}}}} \right)/{\text{d }},$$

where

8 $${\mathbf{E}} = - {\mathbf{X}}_{\text{G}} {\mathbf{D}}^{{\prime }} + {\mathbf{JD}}^{{\prime }} - {\mathbf{DX}}_{\text{G}}^{{\prime }} + {\mathbf{DJ}}^{{\prime }} + {\mathbf{DD}}^{{\prime }} ,$$

and

9 $${\mathbf{J}}_{\text{am}} {\mathbf{J}}_{\text{am}}^{{\prime }} = {\text{m}}{\mathbf{J}}_{\text{aa}} ,$$

$${\mathbf{G}} = \left( {{\mathbf{X}}_{\text{G}} {\mathbf{X}}_{\text{G}}^{\prime } + {\text{m}}{\mathbf{J}}_{\text{aa}} - {\mathbf{X}}_{\text{G}} {\mathbf{J}}_{\text{am}}^{\prime } - {\mathbf{J}}_{\text{am}} {\mathbf{X}}_{\text{G}}^{\prime } + {\mathbf{E}}} \right)/{\text{d }}.$$

10 $${\mathbf{G}}_{\text{u}} = \left( {{\mathbf{X}}_{\text{G}} - {\mathbf{J}}_{\text{am}} } \right)\left( {{\mathbf{X}}_{\text{G}} - {\mathbf{J}}_{\text{am}} } \right)^{\prime } /{\text{d,}}$$

$${\mathbf{G}}_{\text{u}} = \left( {{\mathbf{X}}_{\text{G}} {\mathbf{X}}_{\text{G}}^{\prime } + {\mathbf{J}}_{\text{am}} {\mathbf{J}}_{\text{am}}^{\prime } - {\mathbf{X}}_{\text{G}} {\mathbf{J}}_{\text{am}}^{\prime } - {\mathbf{J}}_{\text{am}} {\mathbf{X}}_{\text{G}}^{\prime } } \right)/{\text{d,}}$$

11 $${\mathbf{G}}_{\text{u}} = \left( {{\mathbf{X}}_{\text{G}} {\mathbf{X}}_{\text{G}}^{\prime } + {\text{m}}{\mathbf{J}}_{\text{aa}} - {\mathbf{X}}_{\text{G}} {\mathbf{J}}_{\text{am}}^{\prime } - {\mathbf{J}}_{\text{am}} {\mathbf{X}}_{\text{G}} } \right)/{\text{d}},$$

$${\mathbf{H}}_{*,1} = \left( {\left( {{\mathbf{K}}\left( {{\mathbf{X}}_{\text{H}} {\mathbf{X}}_{\text{H}}^{\prime } + \left( {{\mathbf{X}}_{\text{H}} - {\mathbf{J}}_{\text{hm}} } \right)\left( {{\mathbf{X}}_{\text{H}} - {\mathbf{J}}_{\text{hm}} } \right)^{\prime } } \right){\mathbf{K}}^{\prime } } \right)/2} \right)/{\text{m,}}$$

$${\mathbf{H}}_{*,1} = (\left( {{\mathbf{KX}}_{\text{H}} {\mathbf{X}}_{\text{H}}^{\prime } {\mathbf{K}}^{\prime } } \right)/2 + \left( {{\mathbf{KX}}_{\text{H}} {\mathbf{X}}_{\text{H}}^{\prime } {\mathbf{K}}^{\prime } } \right)/2 + \left( {{\mathbf{KJ}}_{\text{hm}} {\mathbf{J}}_{\text{hm}}^{\prime } {\mathbf{K}}^{\prime } } \right)/2 - \left( {{\mathbf{KX}}_{\text{H}} {\mathbf{J}}_{\text{hm}}^{\prime } {\mathbf{K}}^{\prime } } \right)/2 - \left( {{\mathbf{KJ}}_{\text{hm}} {\mathbf{X}}_{\text{H}}^{\prime } {\mathbf{K}}^{\prime } } \right)/2)/{\text{m}} ,$$

$${\mathbf{H}}_{*,1} = {\mathbf{KX}}_{\text{H}} {\mathbf{X}}_{\text{H}}^{\prime } {\mathbf{K}}^{\prime } + \left( {{\mathbf{KJ}}_{\text{hm}} {\mathbf{J}}_{\text{hm}}^{'} {\mathbf{K}} '} \right)/2 - \left( {{\mathbf{KX}}_{\text{H}} {\mathbf{J}}_{\text{hm}}^{\prime } {\mathbf{K}}^{\prime } } \right)/2 - \left( {{\mathbf{KJ}}_{\text{hm}} {\mathbf{X}}_{\text{H}}^{\prime } {\mathbf{K}}^{\prime } } \right)/2/{\text{m,}}$$

and since ${\mathbf{X}}_{\text{G}} = {\mathbf{KX}}_{\text{H}}$ and ${\mathbf{KJ}}_{\text{hm}} = 2{\mathbf{J}}_{\text{am}}$,

$${\mathbf{H}}_{ *,1} = \left( {{\mathbf{X}}_{\text{G}} {\mathbf{X}}_{\text{G}}^{\prime } + 2{\mathbf{J}}_{\text{am}} {\mathbf{J}}_{\text{am}}^{\prime } - {\mathbf{X}}_{\text{G}} {\mathbf{J}}_{\text{am}}^{\prime } - {\mathbf{J}}_{\text{am}} {\mathbf{X}}_{\text{G}}^{\prime } } \right)/{\text{m,}}$$

$${\mathbf{H}}_{ *,1} = \left( {{\mathbf{X}}_{\text{G}} {\mathbf{X}}_{\text{G}}^{\prime } + 2{\text{m}}{\mathbf{J}}_{\text{aa}} - {\mathbf{X}}_{\text{G}} {\mathbf{J}}_{\text{am}}^{\prime } - {\mathbf{J}}_{\text{am}} {\mathbf{X}}_{\text{G}}^{\prime } } \right)/{\text{m}} .$$

From Eqs. (10) and (11):

12 $${\mathbf{G}}_{\text{u}} = {\text{m}}\left( {{\mathbf{H}} - {\mathbf{J}}_{\text{aa}} } \right)/{\text{d}} .$$

From Eqs. (7) and (10):

13 $${\mathbf{G}} = {\mathbf{G}}_{\text{u}} + {\mathbf{E}}/{\text{d}} .$$

And finally,

14 $${\mathbf{G}}_{\text{u}} + {\mathbf{E}}/{\text{d}} = \left( {{\text{m}}\left( {{\mathbf{H}} - {\mathbf{J}}_{\text{aa}} } \right) + {\mathbf{E}}} \right)/{\text{d}} .$$

As a result, the extension of ${\mathbf{H}}_{ *,1}$ (Eq. 11) produced the same result as the molecular coancestry suggested by Toro et al. [23].

### Haplotype relationship matrices

**Fig. 1:**
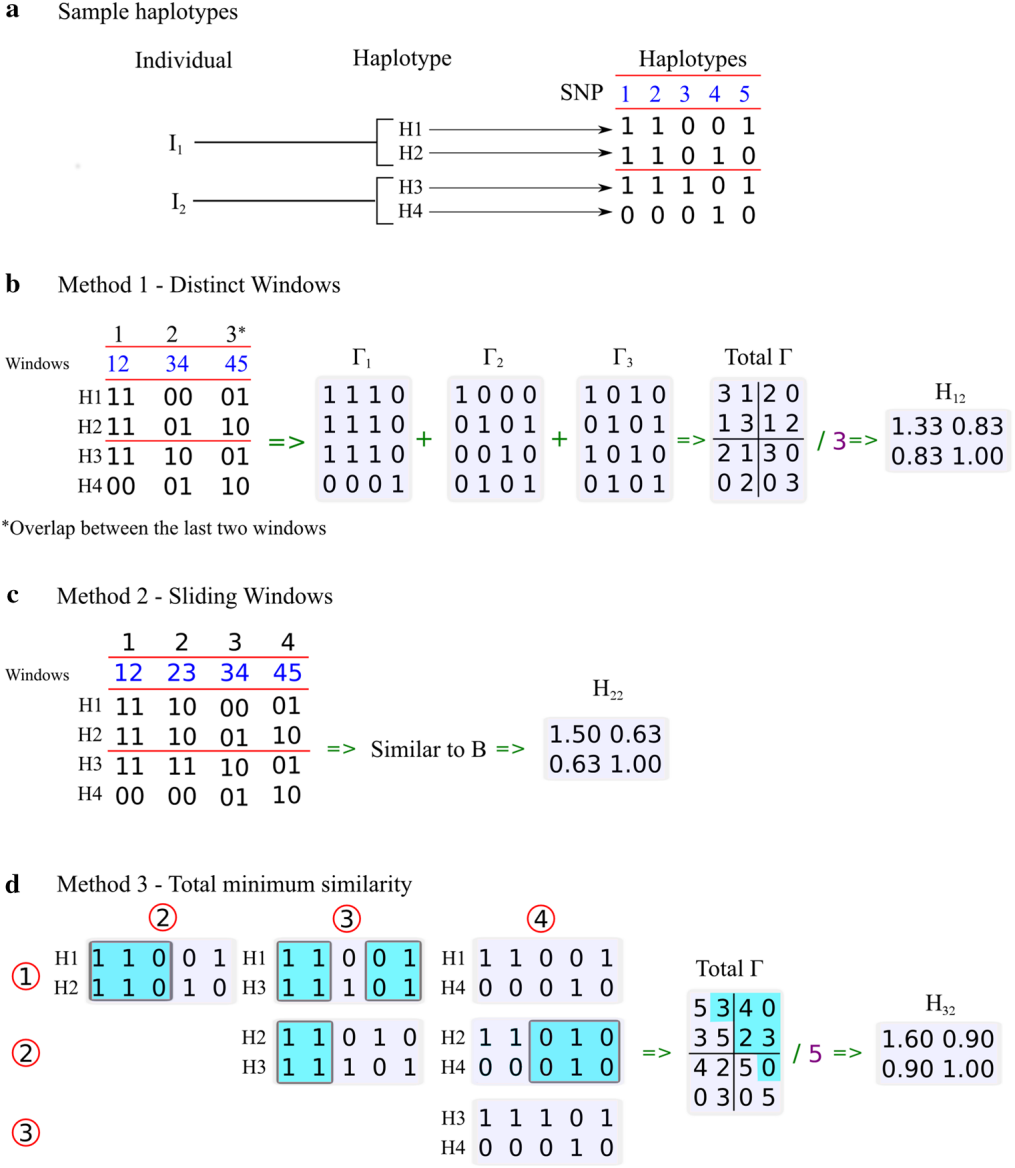
Description of the methods used to build relationship matrices. **a** Haplotypes of five SNPs for two individuals. **b** Method DW, ${\mathbf{H}}_{1,2}$. Each haplotype segment has the same length (${\text{j}} = 2$, ${\text{k}}/{\text{j}} = 5/2 = 3$, ${\text{k}}$ SNPs and window of size ${\text{j}}$), with the last window potentially using SNPs that may have been used in the penultimate segment. ${{\varvec{\Gamma}}}_{\text{i}}$ is the relationship matrix for each window and ${{\varvec{\Gamma}}}$ is the final relationship matrix. **c** Method SW, ${\mathbf{H}}_{2,2}$. Haplotypes that are two SNPs long are constructed from adjacent pairs of SNPs with SNPs present in more than one segment (${\text{j}} = 2$, ${\text{k }} - {\text{j }} + 1 = 5 - 2 + 1 = 4$). **d** Method TMS, ${\mathbf{H}}_{3,2}$. The total number of SNPs in contiguous segments that are identical in pairs of haplotypes. The segment size 2 defines the minimum number of SNPs in two contiguous haplotype segments to be considered as identical by descent (IBD)

Relationships among individuals were calculated in different ways. ${\mathbf{G}}$ [3] was calculated to provide the base to which the three methods were compared. The haplotype relationship matrices in the methods based on different lengths of haplotypes are designated as ${\mathbf{H}}_{{{\text{i}},{\text{j}}}}$ where ${\text{i}}$ is method (1), (2) or (3) (see below and Fig. 1) and ${\text{j}}$ is the length of the haplotypes (${\text{j}}$ = 1, 2, 3, …, 20, 40, 80 and 100). The three methods used to calculate relationships based on haplotypes are illustrated in Fig. 1.

In method (1) or DW for distinct windows, which was used to construct ${\mathbf{H}}_{1,*}$, each chromosome was divided into segments of length ${\text{j}}$. This method was similar to that described by Hickey et al. [15] for building ${\mathbf{H}}_{1}$. A chromosome with ${\text{k}}$ SNPs was divided into ${\text{k }}/ {\text{j}}$ segments so that each SNP appeared only once in any segment (Fig. 1b). Then, in the last segment of a chromosome that was shorter than the segment length, SNPs from the previous segment were included so that all segments had the same length (Fig. 1b).

The gametic relationship matrix (${{\varvec{\Gamma}}}_{\text{segment}}$) among all pairs of haplotypes was determined for each segment by assuming that it was equal to 1 when two haplotypes were the same and 0 when they were not (Fig. 1b).

The gametic relationship matrices for each segment were summed to give a complete gametic relationship matrix (Fig. 1b). The relationship matrix for the whole genome was calculated as follows:

15 $${{\varvec{\Gamma}}} = \mathop \sum \limits_{{{\text{i}} = 1}}^{\text{n}} {{\varvec{\Gamma}}}_{\text{i}} /{\text{n}} ,$$

where ${\text{n}}$ is the number of segments.

This was converted to a relationship matrix at the animal level using (Fig. 1b):

16 $${\mathbf{H}}_{1,1} = {\mathbf{K}} {\varvec{\Gamma}} {\mathbf{K}}^{\prime}/2 .$$

Method (2) or SW for sliding windows was used to construct ${\mathbf{H}}_{2,*}$, which was similar to ${\mathbf{H}}_{1,*}$ but the genome was divided into segments in a different way. In this method, the genome was partitioned into ${\text{k }}{-} {\text{j }} + 1$ segments. The first segment had SNP $1$ to ${\text{j}}$, the second segment had SNP $2$ to ${\text{j }} + 1$ and, so on, to the last segment with SNP ${\text{k }}{-} {\text{j }} + 1$ to ${\text{k}}$ (Fig. 1c).

In method (3) or TMS for total minimum similarity that was used to construct ${\mathbf{H}}_{3,*}$, haplotypes for whole chromosomes were considered. With this method, the number of SNPs in identical segments of length ${\text{j}}$ or more in pairs of haplotypes were counted. These scores were divided by the numbers of SNPs on the chromosome (Fig. 1d).

### Variance components

The model used to analyze the traits was as follows:

$${\mathbf{y}} = {\mathbf{X}}_{{\mathbf{v}}} {\mathbf{b}} + {\mathbf{Z}}_{{\mathbf{v}}} {\mathbf{u}} + {\mathbf{e}},$$

where ${\mathbf{y}}$, ${\mathbf{b}}$, ${\mathbf{u}}$ and ${\mathbf{e}}$ are vectors of observations, fixed effects, breeding values and residuals, respectively, and ${\mathbf{X}}_{{\mathbf{v}}}$ and ${\mathbf{Z}}_{{\mathbf{v}}}$ are design matrices relating observations to effects. ${\text{Var}}\left( {\mathbf{u}} \right) = {\mathbf{W}}{{\upsigma }}_{\text{a}}^{2}$, where ${\mathbf{W}}$ is a relationship matrix which could be ${\mathbf{G}, \mathbf{G}_\text{u}}$ or ${\mathbf{H}}_{{{\text{i}},{\text{j}}}}$. The residual variance was ${\text{Var}}\left( {\mathbf{e}} \right) = {\mathbf{I}}{{\upsigma }}_{\text{e}}^{2}$. Variance components and the log-likelihoods were estimated using ASReml-R version 3 [24].

#### Simulated data

Variance components were estimated for ${\mathbf{G}}_{\text{u}}$, which was similar to ${\mathbf{G}}$ but uncentered, and ${\mathbf{H}}_{1,1}$. In all cases, the only fixed effect (${\mathbf{b}}$) was the mean.

#### Real data

Relationship matrices ${\mathbf{G}}$, and the three ${\mathbf{H}}_{{{\text{i}},{\text{j}}}}$ with varying numbers of loci were used to model covariance between animals. For each method, genetic parameters (i.e. additive and residual variance) for SC, WTCL and AGECL were calculated using the standard single-trait animal models. The optimal length for haplotypes was found by using the profiled log-likelihood for each of the three haplotype-based methods.

### Scaling the haplotype relationship matrix for comparison of additive variances

The additive variances (${{\upsigma }}_{\text{a}}^{2}$) of the haplotype relationship matrices were scaled as in Legarra [25]:

17 $$\left( {{\text{tr}}\left( {{\mathbf{H}}_{ *, *} } \right)/{\text{a}} - \left( {{\mathbf{J}}_{{1{\text{h}}}} {\mathbf{H}}_{ *, *} {\mathbf{J}}_{{{\text{h}}1}} } \right)/{\text{a}}^{2} } \right){{\upsigma }}_{\text{a}}^{2} ,$$

where ‘tr’ is the trace of the matrix and ‘a’ is the number of animals.

### Cross-validation

Fivefold cross-validation was used to assess the accuracy of estimated breeding values (EBV). Individuals were grouped into five subsets of approximately equal size with all the progeny of a common sire in one group. EBV were estimated for each of the five subsets using data from the other subsets and compared with their adjusted phenotypes (phenotypes corrected for the fixed effects).

## Results

### Brahman haplotype diversity

**Fig. 2:**
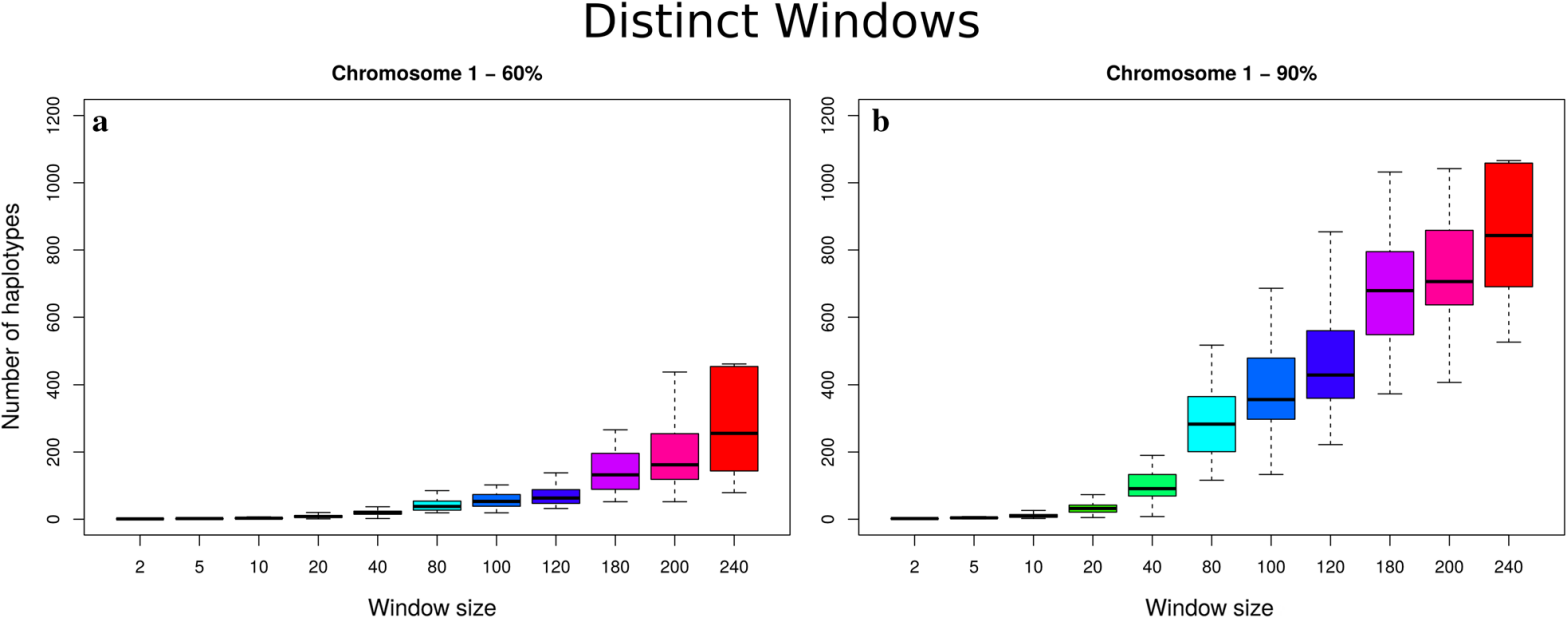
Haplotype diversity for chromosome 1 (Brahman cattle) using two methods and different window sizes for all segments. **a** Number of unique haplotypes required to explain 60 % of the haplotypes. **b** Number of unique haplotypes required to explain 90 % of the haplotypes

Figure 2 shows boxplots for the number of haplotype alleles for all segments that explained 60 and 90 % of the observed haplotype alleles for chromosome 1 using the DW method (similar patterns were observed for the other chromosomes and the SW method—not shown). As the segment size increased, the number of haplotype alleles increased exponentially until the size of the population limited the number of unique haplotypes that could be found.

### Simulated data

Table 2 shows the estimates of variance components when ${\mathbf{G}}$, ${\mathbf{G}}_{\text{u}}$, and ${\mathbf{H}}_{1,1}$ were used in the model. The value of the log-likelihood was similar for all three methods. The mean was somewhat different when ${\mathbf{G}}$ was used compared to that of the other two methods, which share the same mean. The residual variance components were the same when ${\mathbf{G}}_{\text{u}}$ and ${\mathbf{H}}_{1,1}$ were used to describe the covariance between animals, and very similar to the value obtained using ${\mathbf{G}}$. In spite of the differences in the relationship matrices as shown in Eqs. (7–14), the coefficient of determination (R^2^) between elements of the inverses of the different matrices was close to 1. Based on the slope of the regression, the values in ${\mathbf{H}}_{1,1}^{ - 1}$ were nearly two times higher than those in ${\mathbf{G}}^{ - 1}$ and ${\mathbf{G}}_{\text{u}}^{ - 1}$ (ignoring the intercept, see Table 3). Estimated genetic variances were similar when ${\mathbf{G}}$ and ${\mathbf{G}}_{\text{u}}$ were used, but much greater when ${\mathbf{H}}_{1,1}$ was used [26]. The latter result was in agreement with Stranden et al. [27]. As shown in [26], the correlation between the breeding values were close to 1, as were the slopes when any one set of EBV was regressed on any other.

**Table 2:** Log-likelihood, residual variance (${\varvec{\upsigma}}_{{\mathbf{e}}}^{2}$), additive variance (${\varvec{\upsigma}}_{{\mathbf{a}}}^{2}$) and intercept (${\varvec{\upmu}}$) using simulated data and different methods

Method	Log-likelihood	${\varvec{\upsigma}}_{{\mathbf{e}}}^{2}$	${\varvec{\upsigma}}_{{\mathbf{a}}}^{2}$	${\varvec{\upmu}}$
${\mathbf{G}}$	−74.459	36.965	42.377	−5.050
${\mathbf{H}}_{1,1}$	−74.459	37.008	88.654	−5.106
${\mathbf{G}}_{{\mathbf{u}}}$	−74.459	37.007	42.377	−5.106

**Table 3:** Intercept, slope and R^2^ of linear regression of the elements of the inverse relationship matrices from models using different relationship matrices for the simulated data

Dependent variable	${\mathbf{G}}^{ - 1}$	${\mathbf{G}}^{ - 1}$	${\mathbf{H}}_{1,1}^{ - 1}$
Independent variable	${\mathbf{H}}_{1,1}^{ - 1}$	${\mathbf{G}}_{\text{u}}^{ - 1}$	${\mathbf{G}}_{\text{u}}^{ - 1}$
Intercept	34.482	34.470	−0.025
Slope	0.464	0.960	2.069
R^2^	1.000	0.992	0.993

### Real data

#### Haplotype relationship matrices and ${\text{G}}$

**Fig. 3:**
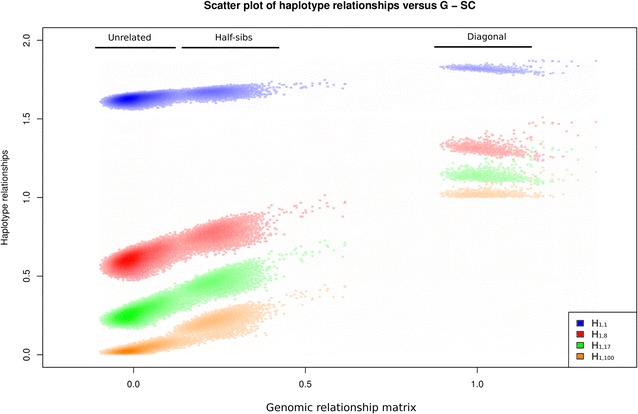
Scatterplot of haplotype relationship matrices for DW and different numbers of SNPs (${\mathbf{H}}_{1,1}$, ${\mathbf{H}}_{1,8}$, ${\mathbf{H}}_{1,17}$, and ${\mathbf{H}}_{1,100}$) versus ${\mathbf{G}}$

Figure 3 shows the scatter plots of the elements ${\mathbf{H}}_{1,1}$, ${\mathbf{H}}_{1,8}$, ${\mathbf{H}}_{1,17}$ and ${\mathbf{H}}_{1,100}$ against the corresponding elements of ${\mathbf{G}}$. The plots for ${\mathbf{H}}_{1, *}$ are in three groups across the X axis (${\mathbf{G}}$), i.e. unrelated, half-sibs and diagonal. In ${\mathbf{G}}$, unrelated individuals have a mean close to 0, half-sibs around 0.23 and the diagonal elements around 1. In ${\mathbf{H}}$, the minimum was 1 in the diagonal elements and 0 in the off-diagonal elements, and the maximum, in both cases, was 2 (Table 4; Fig. 3). However, only the minimum of the diagonal and off-diagonal elements of the relationship matrices with large haplotype segments (${\mathbf{H}}_{1,100}$) reached this minimum limit. The elements of ${\mathbf{H}}_{1,1}$ were much greater than these minimum limits. The mean for the off-diagonal and diagonal elements decreased as segment size increased (Table 4). However, the standard deviations for both off-diagonal and diagonal elements were higher for the intermediate segment sizes (${\mathbf{H}}_{1,8}$ and ${\mathbf{H}}_{1,17}$) than for the very short and long segments (${\mathbf{H}}_{1,1}$ and ${\mathbf{H}}_{1,100}$) (Table 4).

**Table 4:** Minimum, maximum, mean and standard deviation of **G** and **H**_1,*,_ and the correlation of the elements of **G** with the elements of **H**_1*_

	Min	Max	Mean	SD	${\text{r}}_{\text{GU}}$	${\text{r}}_{\text{GH}}$	${\text{r}}_{\text{GD}}$
$${\mathbf{G}}$$							
Off-diagonal	−0.01	0.61	0.00	0.05	1	1	1
Diagonal	0.89	1.34	1.02	0.06			
$${\mathbf{H}}_{1,1}$$							
Off-diagonal	1.56	1.75	1.63	0.02	0.40	0.27	−0.34
Diagonal	1.77	1.88	1.82	0.01			
$${\mathbf{H}}_{1,8}$$							
Off-diagonal	0.47	1.02	0.60	0.04	0.56	0.47	−0.09
Diagonal	1.23	1.51	1.31	0.03			
$${\mathbf{H}}_{1,17}$$							
Off-diagonal	0.16	0.73	0.26	0.04	0.68	0.63	0.12
Diagonal	1.08	1.36	1.14	0.03			
$${\mathbf{H}}_{1,100}$$							
Off-diagonal	0.00	0.44	0.03	0.03	0.71	0.73	0.30
Diagonal	1.00	1.19	1.02	0.02			

${\text{r}}_{\text{GU}}$: correlation between the unrelated individuals (elements of ${\mathbf{G}}$ and ${\mathbf{H}}_{1, *}$); ${\text{r}}_{\text{GH}}$: correlation between the half-sibs individuals (elements of ${\mathbf{G}}$ and ${\mathbf{H}}_{1, *}$); ${\text{r}}_{\text{GD}}$: correlation between the diagonal elements of ${\mathbf{G}}$ and ${\mathbf{H}}_{1, *}$

Correlations between the off-diagonal elements of ${\mathbf{G}}$ and ${\mathbf{H}}_{ *, *}$ were positive. The correlation between off-diagonal elements increased as the segment size increased. However, only the diagonal elements of ${\mathbf{H}}_{1,17}$ and ${\mathbf{H}}_{1,100}$ were positively correlated with the elements of ${\mathbf{G}}$. Although the elements of half-sib individuals were less correlated with ${\mathbf{G}}$ elements than the elements of unrelated individuals, there was a higher correlation between the elements of half-sibs individuals than between the diagonal elements and ${\mathbf{G}}$.

#### Variance components

**Fig. 4:**
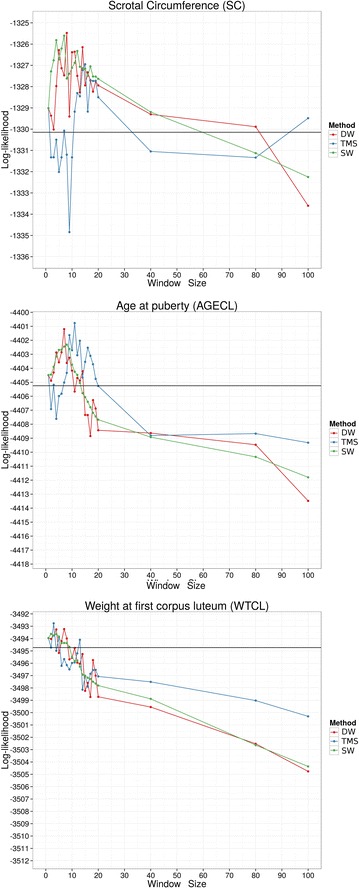
Log-likelihood of the three methods used to build relationship matrices for three traits (SC, AGECL and WTCL) and different window sizes. The *black horizontal line* represents the result obtained when using ${\mathbf{G}}$. *DW* distinct windows, *SW* sliding windows, *TMS* total minimum similarity

The log-likelihoods evaluated for SC, AGECL and WTCL using ASReml-R are in Fig. 4. For all traits, the log-likelihoods of the ${\mathbf{H}}_{ *, *}$ methods were higher than that of ${\mathbf{G}}$ when haplotype length was longer than one-SNP. The three methods gave similar results for all traits. Regardless of the method used for dividing the haplotype, the log-likelihoods decreased as the segment size increased from 10 to 20 SNPs and the log-likelihoods were higher than that of ${\mathbf{G}}$ (black line), except for WTCL. However, the log-likelihood for WTCL increased slightly when the haplotype length was less than 10 SNPs. The best values for each trait are in Table 5.

**Table 5:** Window size, log-likelihood, residual variance (${\varvec{\upsigma}}_{{\mathbf{e}}}^{2}$), additive variance (${\varvec{\upsigma}}_{{\mathbf{a}}}^{2}$) and intercept (${\varvec{\upmu}}$) with the best log-likelihood using real data and different methods

Trait	Method	DW	SW	TMS	${\mathbf{G}}$
Scrotal circumference	Window size	8	7	15	–
	Log-likelihood	−1325.48	−1325.60	−1326.95	−1330.15
	Additive variance	5.29	5.44	5.02	3.20
	Residual variance	2.03	2.11	2.32	2.44
Age at puberty	Window size	7	8	11	–
	Log-likelihood	−4401.20	−4402.31	−4400.77	−4405.25
	Additive variance	11273.73	11052.93	11853.68	6816.86
	Residual variance	4902.01	4694.35	5405.72	5729.85
Weight at first corpus luteum	Window size	7	2	3	–
	Log-likelihood	−3493.23	−3493.62	−3492.76	−3494.73
	Additive variance	1451.34	2758.13	3184.40	900.60
	Residual variance	750.88	804.50	845.43	833.38

**Fig. 5:**
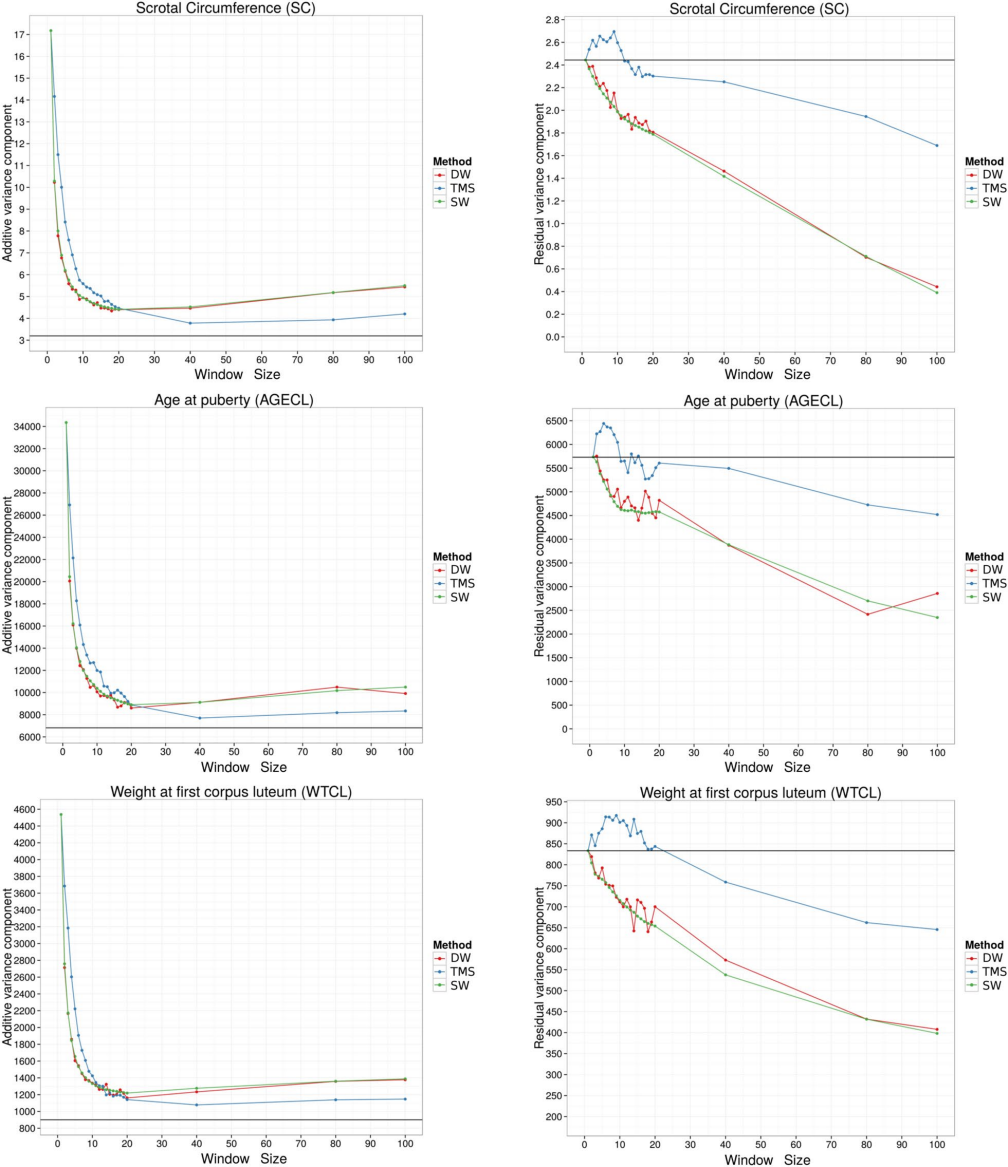
Additive and residual variances for the three methods used to build relationship matrices for three traits (SC, AGECL and WTCL) and different window sizes. The *black horizontal line* represents the ${\mathbf{G}}$ result. *DW* distinct windows, *SW* sliding windows, *TMS* total minimum similarity

The additive and residual variances for each trait are in Fig. 5. For short haplotypes, the additive variance estimated using ${\mathbf{H}}$ was much greater than that estimated using ${\mathbf{G}}$. The additive variance component decreased substantially as the segment length increased to 20 SNPs, but stabilized as it became longer than 20 SNPs. The residual variance decreased considerably as the segment size increased, except for the TMS method. In contrast to the SW and DW methods, the residual component for the TMS method was larger when the segment size was less than 10 SNPs.

**Fig. 6:**
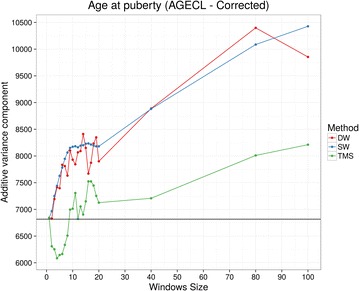
Additive variance for the three methods scaled by using Legarra [25] to build different relationship matrices for AGECL. The *black horizontal line* represents the result obtained when using ${\mathbf{G}}$. *DW* distinct windows, *SW* sliding windows, *TMS* total minimum similarity

The additive variances generated from scaled relationship matrices [25] are in Fig. 6. Contrary to unscaled relationship matrices, the additive variance for the one-SNP relationship matrix was similar to that for ${\mathbf{G}}$ and as window size increased, additive variances increased.

### Cross-validation

**Fig. 7:**
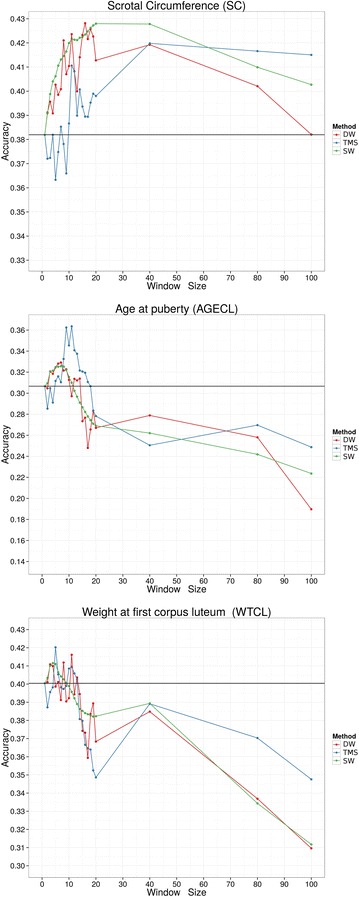
Accuracies of the three methods used to build relationship matrices for three traits (SC, AGECL and WTCL) and different window sizes. The *black horizontal line* represents the result obtained with ${\mathbf{G}}$. *DW* distinct windows, *SW* sliding windows, *TMS* total minimum similarity

The correlation between adjusted phenotypes and EBV increased with the likelihood and number of SNPs per window. The SW method had the highest prediction accuracy for SC, and the TMS method had the highest accuracy for AGECL and WTLCL. However, when we looked at the standard deviations, the differences in accuracy could not be considered as significant. Similar to the log-likelihood, the best length of haplotype was trait-dependent (Fig. 7). The best accuracies for each trait are in Table 6. Except for SC, the window sizes that achieved the highest log-likelihood and accuracy were close to each other.

**Table 6:** Window sizes for the best log-likelihood and accuracies using real datasets (mean ± SD)

Trait	Method	DW-AC	DW-LL	SW-AC	SW-LL	TMS-AC	TMS-LL
Scrotal circumference	Window size	16	8	20	7	40	15
	Accuracy	0.43 ± 0.07	0.42 ± 0.07	0.43 ± 0.09	0.41 ± 0.06	0.42 ± 0.07	0.39 ± 0.06
	**G** accuracy	0.38 ± 0.07					
Age at puberty	Window size	7	7	7	8	11	11
	Accuracy	0.33 ± 0.17	0.33 ± 0.17	0.33 ± 0.18	0.33 ± 0.19	0.36 ± 0.17	0.36 ± 0.17
	**G** accuracy	0.31 ± 15					
Weight at first corpus luteum	Window size	11	7	4	2	5	3
	Accuracy	0.42 ± 0.15	0.40 ± 0.15	0.41 ± 0.13	0.40 ± 0.14	0.42 ± 0.11	0.37 ± 0.14
	**G** accuracy	0.40 ± 14					

*DW* distinct windows, *SW* sliding windows, *TMS* total minimum similarity

AC: window size for the best accuracy, LL: accuracy and windows size for the best log-likelihood

## Discussion

Models based on haplotypes of optimum length to describe relationships among individuals were always better than models using ${\mathbf{G}}$, however the optimum haplotype length depended on the trait. The improvement in log-likelihood resulting from the use of haplotypes rather than one-SNP was most likely due to the LD between SNPs and QTL being better captured (at the intermediate age of the base population [4]), especially at short-range LD [10]. The other reason for the improvement in log-likelihood was that more genetic diversity was captured with haplotypes than with ${\mathbf{G}}$ [3]. Although these two datasets were relatively small, there is no reason to suspect that these results will not extend to the analysis of larger datasets. The results for different methods and traits suggest that haplotypes can make better use of genotype data for genomic prediction. With the real data, the optimum haplotype length was trait-dependent and could be estimated from the data.

Although there was considerable variation between the different haplotype-based methods both across and within the traits with longer haplotypes, the log-likelihood profiles and accuracies increased as the segment size increased up to window size 10, and then both decreased as the segment size increased further (Figs. 4, 7 for AGECL and SC). The decay in log-likelihood occurred because the use of large segments resulted in the relationship matrix tending towards an identity matrix as the variance of the relationships was reduced (Table 4). As a result, the relationships between individuals became closer to zero which makes it difficult to calculate the additive and residual variances. With very long haplotypes, relationships between parent and offspring or between full-sibs were less than 0.5, and between half-sibs less than 0.25. Therefore, using an appropriate method of haplotype partitioning is very important. As the segment size increased, only recent relationships between individuals could be captured and the optimum haplotype length may be an indicator of the optimum age of relationship between individuals [4]. However, for these analyses, there were only minor differences in optimum lengths of haplotype for each trait and method. Using this haplotype-based method in a multiple trait analysis may require the use of different relationship matrices for each trait. If so, then the blocks between animal and traits in the relationship matrix among all breeding values would need to be built and inverted explicitly, thus dramatically increasing the already difficult computational problem for these types of analyses. Nevertheless, there may be a suitable haplotype length that would permit the use of one genomic relationship matrix across all traits. For example, the method based on discrete windows had an optimum of seven SNPs per haplotype for AGECL and WTCL and eight for SC. However there was little difference between the results for SC when the ${\mathbf{H}}_{1,8}$ was used compared to ${\mathbf{H}}_{2,7}$ (Table 5).

A feature of all three haplotype-based methods was that the additive variance was much greater than that found when using ${\mathbf{G}}$, simply because ${\mathbf{H}}_{ *, *}$ and ${\mathbf{G}}$ have different scales (Table 2 and 3). However, the additive variance decreased rapidly as the number of SNPs that form the haplotypes increased (Fig. 5). Hence, it is important to estimate the genetic variance by using the appropriate relationship matrix.

Unlike additive variances, residual variances for ${\mathbf{H}}_{ *, *}$ were generally smaller than those obtained when using ${\mathbf{G}}$, except for the TMS method. Residual variances decreased as the window size increased for the same reason that the log-likelihood decreased, i.e. longer haplotypes resulted in a relationship matrix that was similar to an identity matrix. In the TMS method for small segments, the elements of the residual variances were greater than for ${\mathbf{G}}$ and the other methods. Consequently, this method may not be suitable for capturing the true relationships.

When only one-SNP haplotypes were used, all three methods provided the same ${\mathbf{H}}_{ *,1}$ matrix and subsequent results [26]. As previously noted, the EBV obtained by using ${\mathbf{H}}_{ *,1}$ to describe the relationships were the same as those estimated using ${\mathbf{G}}$. The difference in their means was not important since it did not change relative merit as defined by differences in the breeding values. This occurred although the estimated genetic variances were much higher when ${\mathbf{H}}_{ *,1}$ was used, than when ${\mathbf{G}}$ was used, to model the relationships. Clearly, the effects of using ${\mathbf{G}}$ and ${\mathbf{H}}_{ *,1}$ for estimating breeding values were similar, as were their inverses (Table 3). However, the elements in ${\mathbf{H}}_{ *,1}$ appeared to be on a different scale, being much higher than those observed in ${\mathbf{G}}$. These very high coefficients suggest that the individuals were highly related and inbred, compared to the implied founder population. The scale of the relationship matrices based on genomic data is very important for the computation of heritability and combining genotyped and ungenotyped individuals in the so-called single-step analysis. The EBV [26] clearly indicated that the genotypic information was used in the same way in all methods for prediction. We have demonstrated how a change to ${\mathbf{G}}$, ${\mathbf{G}}_{\text{u}}$ and ${\mathbf{H}}_{ *, *}$ can be directly related to one another, as demonstrated in Eqs. (7–14).

An alternative method for appropriate scaling of the relationships among individuals is necessary. There are three possible methods for scaling. One was developed for scaling ${\mathbf{G}}$ based on the pedigree [28]. A second method, since ${\mathbf{H}}_{1,1}$ demonstrates the molecular coancestry that can be rescaled to genealogical coancestry with the formula in [23], uses a similar formula with a slight modification to rescale ${\mathbf{H}}_{ *, *}$ with segment sizes larger than 1. A third scaling method was suggested by Legarra [25] for scaling the relationship matrices in order to compare their additive variances. However, further research is required to identify which of these methods provide the most accurate scaling of the haplotype relationship matrix.

The optimum haplotype lengths to achieve the highest accuracy and log-likelihood were similar for AGECL and WTCL whereas for SC the optimum haplotype length for each method varied considerably. This may be caused by the high heritability of the SC trait, although the difference in improvement of accuracy for both optimum lengths was not significant (Table 6).

Only three methods for building haplotype-based relationships were used in this paper. Other methods to create the haplotypes or relationships may improve the accuracy. Two obvious methods that were not tested in this paper are based on the physical position of markers or the linkage maps of the genome. Alternatively, a more complete approach to modeling relationships between haplotypes within each segment would include non-zero correlations between haplotypes. Such correlations would be based on methods that estimate the evolutionary relationships among haplotypes.

In addition, the effect of heritability and genotyping errors should be considered when comparing the improvement in accuracy and log-likelihood of different traits. Simulation studies have shown that using haplotype segments can increase the accuracy of genomic selection for traits with a high heritability [11]. However, the effect of heritability on the accuracy should be checked with real data. In the current study, the effect of heritability on the increase in accuracy and log-likelihood profile was observed for SC with a heritability of 0.75, which led to a high accuracy even when large haplotype segments were used (Table 6). Moreover, genotyping and haplotype reconstruction errors should be considered when building the relationship matrix. These errors may be one of the reasons that explain the fluctuation in accuracy and log-likelihood observed in this paper for different window sizes, especially with the DW method, which is more sensitive to this kind of issue. In addition, the rate of the decrease in prediction accuracy as segment size increases would be affected by these errors, i.e. genotyping errors will cause more problems for large segments than for small segments.

## Conclusions

In this article, three strategies to build relationship matrices using haplotype segments were evaluated. When one-SNP haplotypes are used, we showed and proved that the current methods and the ${\mathbf{G}}$ matrix of VanRaden [3] were the same but on different scales. In addition, using more than one-SNP as a haplotype segment can improve the log-likelihood of genomic selection. For example, the log-likelihood of SC with ${\mathbf{H}}_{1,8}$ was increased by 4.67 in comparison to that with the ${\mathbf{G}}$ matrix of VanRaden [3] which was equal to −1330. The optimum haplotype length varied and depended on the methods used for creating relationship matrices, as well as the traits studied, and varied also across datasets. Hence, other methods for haplotype partitioning based on the linkage map or smooth correlation between haplotype segments may improve the prediction accuracy.
